# Diet quality across early childhood and adiposity at 6 years: the Southampton Women's Survey

**DOI:** 10.1038/ijo.2015.97

**Published:** 2015-06-30

**Authors:** H Okubo, S R Crozier, N C Harvey, K M Godfrey, H M Inskip, C Cooper, S M Robinson, SWS Study Group

**Affiliations:** 1 MRC Lifecourse Epidemiology Unit, Southampton General Hospital, University of Southampton, Southampton, UK; 2 Department of Health Promotion, National Institute of Public Health, Saitama, Japan; 3 National Institute of Health Research, Southampton Biomedical Research Centre, University of Southampton and University Hospital Southampton National Health Service Foundation Trust, Southampton, UK; 4 National Institute for Health Research Musculoskeletal Biomedical Research Unit, Nuffield Orthopedic Centre, University of Oxford, Oxford, UK

## Abstract

**Background:**

Poor diet quality in early childhood is inconsistently linked to obesity risk. Understanding may be limited by the use of cross-sectional data and the use of body mass index (BMI) to define adiposity in childhood.

**Objective:**

The objective of this study is to examine the effects of continued exposure to diets of varying quality across early childhood in relation to adiposity at 6 years.

**Methods:**

One thousand and eighteen children from a prospective UK birth cohort were studied. Diet was assessed using food frequency questionnaires when the children were aged 6 and 12 months, and 3 and 6 years; diet quality was determined according to scores for a principal component analysis-defined dietary pattern at each age (characterized by frequent consumption of fruits, vegetables and fish). At each age, children were allocated a value of 0/1/2 according to third of the distribution (bottom/middle/top) their diet quality score was in; values were summed to calculate an overall diet quality index (DQI) for early childhood (range 0–8). Obesity outcomes considered at 6 years were dual-energy X-ray absorptiometry-assessed fat mass and BMI.

**Results:**

One hundred and seven (11%) children had a DQI=0, indicating a consistently low diet quality, 339 (33%) had a DQI=1–3, 378 (37%) had a DQI=4–6 and 194 (19%) had a DQI=7–8. There was a strong association between lower DQI and higher fat mass *z*-score at 6 years that was robust to adjustment for confounders (fat mass s.d. per 1-unit DQI increase: *β*=−0.05 (95% confidence interval (CI): −0.09, −0.01), *P*=0.01). In comparison with children who had the highest diet quality (DQI=7–8), this amounted to a difference in fat mass of 14% (95% CI: 2%, 28%) at 6 years for children with the poorest diets (DQI=0). In contrast, no independent associations were observed between DQI and BMI.

**Conclusions:**

Continued exposure to diets of low quality across early childhood is linked to adiposity at the age of 6 years.

## Introduction

The prevalence of overweight and obesity in children is increasing rapidly and now is one of the most serious public health challenges worldwide.^1^ Excess weight not only has implications for health in childhood^2, 3^ but is also linked to an increased risk of adult obesity and its related adverse metabolic consequences in later life.^4^ As differences in body composition are evident from early childhood, successful preventive strategies in the future will rely on understanding the role of modifiable factors in early life and their influence on the child's risk of obesity.^5^

There is a growing interest in the role of diet quality in early childhood as a determinant of obesity risk.^6, 7, 8^ Diets of poor quality might be expected to be important, as they are often characterized by high consumption of energy-dense foods^9, 10^ and there is growing evidence that food intake patterns and dietary behaviors established in early childhood ‘track' into later life.^11^ Recent systematic reviews have explored associations between diet quality in early life and health-related outcomes.^6, 7, 8, 12^ Weight status and risk of overweight/obesity were the most commonly assessed health-related outcomes, but surprisingly, findings in relation to diet quality and childhood obesity are not consistent across studies.^8^ One possibility is that much of the evidence is based on cross-sectional comparisons, whereas longitudinal data are needed to address the cumulative effects of exposure to diets of poor quality. Another issue may be the limitation of using body mass index (BMI) as an indicator of adiposity in children.^13^

In this study we describe the relations of diet quality at four ages from infancy to early childhood, with body composition and obesity risk at 6 years, among 1018 children who participated in a prospective birth cohort, the Southampton Women's Survey (SWS). We consider the associations with a diet quality index (DQI), that describes overall exposure to diets of varying quality across early childhood, and diet quality at each age, in relation to dual-energy X-ray absorptiometry (DXA)-assessed measures of fat mass and BMI.

## Subjects and methods

### The Southampton Women's Survey

The SWS study is an ongoing, prospective cohort study of 12 583, initially non-pregnant, women aged 20–34 years, living in the city of Southampton, UK.^14^ Assessments of lifestyle, diet and anthropometry were performed at study entry (April 1998–December 2002). Women in the SWS study, who subsequently became pregnant, were followed up at 11, 19 and 34 weeks of gestation; the offsprings have been studied in infancy and childhood.

The SWS study was conducted according to the guidelines laid down in the Declaration of Helsinki and was approved by the Southampton and South West Hampshire Local Research Ethics Committee (06/Q1702/104). Written informed consent was obtained from all participating women and by a parent or guardian with parental responsibility on behalf of their children.

### Dietary assessment in infancy and childhood

Diet was assessed when the children were aged 6 and 12 months, and 3 and 6 years, using age-specific validated questionnaires that were administered to the child's parent or guardian by trained research nurses.^15, 16, 17^ At 6 months of age, food intake over the previous 7 days was assessed using a 34-item food frequency questionnaire (FFQ).^15^ At 12 months of age, food intake over the previous 4 weeks was assessed using a 78-item FFQ.^16^ At 3 and 6 years of age, diet over the preceding 3 months was assessed using an 80-item FFQ.^17^ The average frequency of consumption of the listed foods was recorded. At all interviews, prompt cards were used to show the foods included in each food group, to ensure standardized responses to the FFQ. At the end of each FFQ, an open section in the same format was included, to record consumption frequencies of any foods that were not listed on the FFQ, if they were consumed once per week or more. Daily volumes and types of milks consumed and amount of sugar added to foods were recorded.

The dietary patterns of children in the SWS study were identified at each age using principal component analysis (PCA). Before the PCA analysis, the foods listed on the FFQ were grouped on the basis of similarity of type of food and nutrient composition; a total of 46 groups at 6 months, 56 groups at 12 months and 51 groups at 3 and 6 years were entered into the PCA analysis. More detailed descriptions of dietary patterns in infancy and childhood have been published elsewhere.^9, 18^ The first component at each age, which described the greatest variance in the dietary data, was a ‘healthy' dietary pattern that complied with dietary recommendations, characterized by frequent consumption of fruit, vegetables and fish.^9, 18^ We named this an ‘infant guidelines' pattern at 6 and 12 months and a ‘prudent' dietary pattern at 3 and 6 years; the coefficients for the prudent component in the PCA analysis at 6 years are shown in Supplementary Table S1. Infant guidelines and prudent diet scores were calculated for every child using the pattern coefficients for each food/group on the FFQ together with their reported frequency of consumption at the respective ages. The scores describe compliance with the infant guidelines/prudent dietary patterns and were used as an indicator of the quality of the children's diets at each age. We refer to them as a ‘diet quality score' at each of the four ages included in this study.

The relative validity of the estimates of intake from the FFQs was established in comparison with intakes assessed using prospective food diaries in validation studies carried out at 6 months, 12 months and 3 years.^15, 16, 17^ There was no separate validation study at 6 years. Energy-adjusted Spearman's correlation coefficients for macro- and micronutrient intake ranged between 0.55 and 0.89 at 6 months (median 0.74),^15^ 0.24–0.75 (median 0.5) at 12 months^16^ and 0.41–0.59 (median 0.51) at 3 years.^17^ PCA analysis was carried out using a 24-h recall data at 6 months and food diary data at 3 years; Spearman's correlation coefficients for the first component (infant guidelines at 6 months and prudent diet at 3 years) scores were 0.81^(ref. 18)^ and 0.72,^17^ respectively.

### Infancy and childhood data, and assessment of body composition

At birth, the baby was weighed on calibrated digital scales (Seca, Birmingham, UK). Duration of breastfeeding was defined according to the date of the last breastfeed recorded at the 6- and 12-month visits.^19^ When the children were aged 4 years, physical activity level was determined according to the child's average number of hours spent ‘on the move' and screen time (TV or computer) each day, as reported by their parent.^20^ Average time for sleeping per night was assessed using information provided by the parents about the child's usual bedtime, an estimate of how long they were awake for during the night and usual time of rising. In addition, parents were asked about the duration of any naps during the day. At a home visit when the children were aged 6 years, height (cm) was measured with the use of a portable stadiometer (Leicester height measure; Seca); weight (kg) was measured using calibrated digital scales (Seca). These data were used to calculate children's BMI. The children who completed the home visit survey (*n*=1103) were subsequently invited to clinic for an assessment of body composition by DXA. Total and proportionate fat mass were derived from the whole-body scan through the use of pediatric software. The total X-ray dose for the whole-body scans were ~10.5 microsieverts (pediatric scan mode), which is equivalent to ~1- to 2-day background radiation. All scan results were checked independently by two trained operators. The coefficient of variation for body composition analysis using the DXA instrument was 1.4–1.9%.

### Maternal data

Details of maternal educational attainment (defined in six groups according to highest academic qualification)^21^ and parity were obtained during the pre-pregnant interviews. Height and weight were measured at this visit and these measurements were used to calculate prepregnancy BMI. Among women who became pregnant, smoking status in pregnancy was ascertained at the 11- and 34-week interviews. At 34 weeks of gestation, the research nurses weighed the women again. In previous analyses of body composition data in this cohort,^22, 23^ we have described associations between maternal vitamin D status and plasma fatty acid concentrations with offspring adiposity at 6 years. We therefore considered these variables as potential determinants of adiposity and included them in the statistical models. A venous blood sample was taken into heparinized tubes at 34 weeks of gestation and an aliquot of maternal serum was frozen at −80 °C. Serum 25-hydroxyvitamin D concentration was analyzed by radioimmunoassay (Diasorin, Stillwater, MN, USA). This assay measures both 25-hydroxyvitamin D2 and D3. The assay met the requirements of the UK National Vitamin D External Quality Assurance Scheme; intra-assay and inter-assay coefficients of variation were <10%. Fatty acid composition of maternal plasma phosphatidylcholine was also measured as described previously.^24^

### Statistical analysis

A total of 1852 women became pregnant and delivered healthy, term (after 37 weeks' gestation) singleton infant, up to the end of 2003. Of these mother–child pairs, 834 were not included in the analyses, either because they did not have body composition data at 6 years or dietary data for at least two ages in early childhood. Data for 1018 children were presented; 725 (71%) children had complete dietary data at all four ages.

Descriptive data are presented as mean (s.d.) or median (interquartile range) for continuous variables and percentages of subjects for categorical variables. To investigate whether there was evidence of a cumulative effect of continued exposure to diets of varying quality across early childhood and a single effect of diet quality at each of the four ages, an index of overall diet quality was derived. The distributions of diet quality scores at each of the four ages were categorized into thirds. Each child was assigned a value of 0, 1 or 2 according to where their diet quality score was in the distribution at each age: 0 (lowest), 1 (middle) and 2 (highest). The values were summed to yield a DQI across early childhood, ranging from 0 (lowest diet quality across early childhood) to 8 (highest diet quality across early childhood). For children who did not have four dietary assessments (42 children had 2 and 251 had 3), the average value from their assessments was substituted for the missing value. The DQI across early childhood was used both as a continuous score and also as a categorical (grouped as 0, 1–3, 4–6 and 7–8) variable. Spearman's correlation was used to describe the associations between diet quality scores at each of the four ages and the relations with the DQI across early childhood. Differences in selected characteristics according to the DQI were examined using a linear model.

Univariate and multiple linear regression analyses were performed to explore the associations of diet quality scores at each of the four ages and the DQI across early childhood with BMI and body fat mass at 6 years. Children's BMI at 6 years was adjusted for sex and age at measurement using a regression model; children's fat mass at 6 years was adjusted for sex, age and height using a regression model, the adjustment for height being necessary to ensure that any associations were independent of children's stature. These adjusted outcomes were used throughout the unadjusted (Model 1) and adjusted (Model 2) analyses. All outcomes were positively skewed and thus were log-transformed and internally standardized (mean±s.d.; 0±1) for ease of interpretation. In the regression analyses (Model 2), we also considered associations between diet quality and body composition outcomes after taking account of a number of potential confounding factors; maternal factors were prepregnancy BMI, Institute of Medicine weight-gain categories,^25^ smoking status in pregnancy, vitamin D status (serum 25-hydroxyvitamin D) at 34 weeks of gestation^22^ and plasma *n*-6 polyunsaturated fatty acid status at 34 weeks of gestation;^23^ offspring factors were duration of breastfeeding, average time ‘on the move' at 4 years (h per day) and time spent watching TV or on the computer at 4 years (h per day). In addition, in order to provide measures of effect of diet quality score on fat mass, we back transformed the effect sizes calculated using the logged fat mass data (by exponentiating the estimates) to describe differences in fat mass as measured (in kilograms); these are expressed as percentage differences. Further analyses with binary outcomes for risk of overweight/obesity used Poisson regression with robust variance to calculate relative risks.^26^ Where dietary data were categorized into thirds of diet quality scores at each age and four groups of the DQI, the highest category was used as a reference group.

All statistical analyses were performed using Stata version 13.1 (Statacorp LP, College Station, TX, USA).

## Results

### Characteristics of study population

The children included in the analyses were more likely to have been breastfed for longer than other SWS children (Table 1). Compared with the other mothers in the SWS cohort (*n*=834), those included in the analyses (*n*=1018, 55% of all live births) were slightly older at delivery and taller, and they tended to have a higher level of educational attainment, to be primiparous, and to be less likely to smoke in pregnancy (all *P*<0.01). There were no differences in gestational age, child's birthweight, maternal prepregnancy BMI, maternal serum 25-hydroxyvitamin D concentration, plasma *n*-6 polyunsaturated fatty acid concentration in late pregnancy or Institute of Medicine weight-gain categories between mother–child pairs studied and the remaining pairs.

### Diet quality score in infancy and childhood according to the DQI

The correlation matrix for the diet quality scores at each age is shown in Supplementary Table S2. Diet quality scores were correlated at all ages (range of Spearman's correlation coefficients, *r*=0.32–0.69). Although the strongest correlations were observed in assessments carried out at the closest ages, diet quality at 6 months of age was associated with diet quality at 6 years (*r*=0.32), indicating tracking in diet from early infancy.

**Figure 1:**
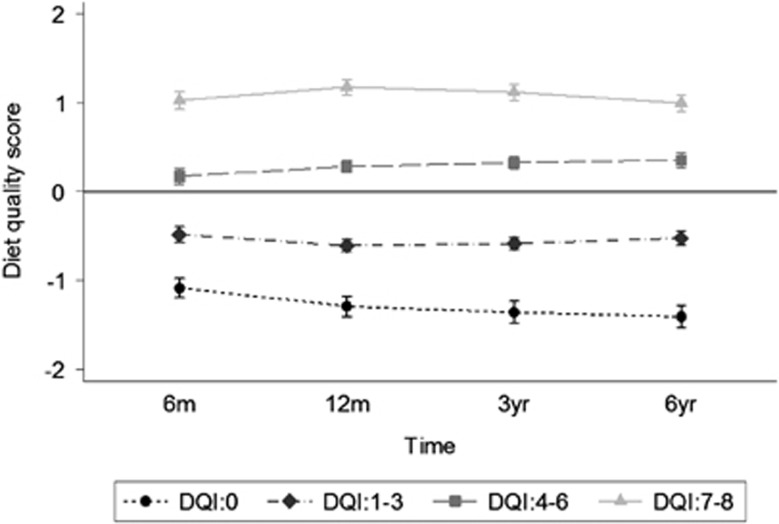
Mean (and 95% CI) diet quality score at each age according to the group of the DQI across early childhood. Diet quality score on *y*-axis describes scores of an infant guidelines pattern at 6 and 12 months, and a prudent dietary pattern at 3 and 6 years, which were determined by a PCA analysis at each age. The scores indicate compliance with the infant guidelines/prudent dietary patterns (characterized by frequent consumption of fruits, vegetables and fish) at each age assessed according to the group of the DQI across early childhood.

When the children were classified into four groups according to the DQI across early childhood, 107 (10.5%) of the children had the lowest diet quality across early childhood (that is, DQI=0), 339 (33.3%) had a DQI=1–3, 378 (37.1%) had a DQI=4–6 and 194 (19.1%) consistently had a high diet quality across early childhood (DQI=7–8). Mean diet quality scores assessed at each age according to DQI group are shown in Figure 1. This demonstrated that the DQI groups clearly separated the children in terms of average diet quality at each age. It is a concern that there was a sizeable group of children (11%) categorized into the lowest DQI group who consistently had the poorest quality diets, in the lowest third of diet quality scores, at each of the four ages.

### Characteristics of study population according to the DQI

Across the distribution of DQI, there were differences in mother–child characteristics, although there were no differences in the proportion of boys and girls across the groups, or in height and weight of the children at 6 years (Table 2). Children with a higher DQI were likely to have been breastfed for longer (all *P*<0.01) and to spend less time watching television at 4 years, although unexpectedly they were also less likely to ‘be on the move' each day. Higher DQI was associated with older mothers who had greater levels of education and lower prepregnancy BMI, and who were less likely to have smoked and who had higher vitamin D status in pregnancy than other mothers.

### Associations of diet quality in early childhood with adiposity at 6 years

Inverse associations were observed between diet quality scores at all four ages in relation to fat mass at 6 years (all *P*<0.001; Table 3). With the exception of the association at 12 months of age, these associations remained after adjustment for maternal and child factors (all *P*=0.01). In contrast, diet quality score at 6 and 12 months, and 3 years were inversely associated with BMI at 6 years, but these associations were no longer apparent after adjustment for potential confounding factors. There was no cross-sectional association between diet quality score at 6 years and BMI.

**Figure 2:**
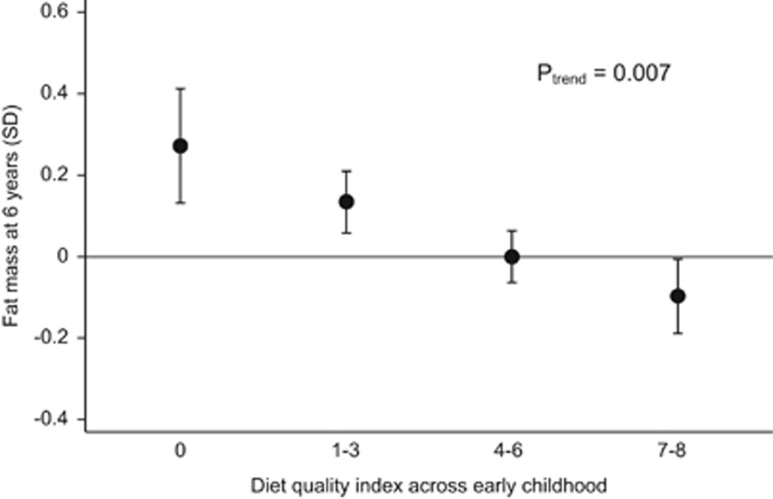
Mean (and 95% CI) fat mass at 6 years according to four groups of DQI across early childhood. Fat mass (s.d.) at 6 years were adjusted for sex, age at measurements, child's height, maternal prepregnancy BMI, Institute of Medicine (IOM) weight-gain categories, maternal smoking in pregnancy, maternal serum 25-hydroxy vitamin D concentration at late pregnancy, maternal plasma *n*-6 polyunsaturated fatty acid concentration at late pregnancy, duration of breastfeeding, time moving each day at 4 years and time spent watching TV at 4 years.

There was a strong inverse association between DQI across early childhood and fat mass at 6 years, which was evident before and after adjustment for confounding factors. This is illustrated in Figure 2, where the graded relation between DQI and fat mass (s.d.) is clear. In comparison with the children who had the highest diet quality across early childhood (DQI=7–8), the size of the difference in fat mass (kg) at 6 years was 8% (95% confidence interval (CI): 1%, 17%) greater among the children whose DQI was 1–3 and 14% (95% CI: 2%, 28%) greater among the children who consistently had a low diet quality across early childhood (DQI=0). In contrast, no independent association was observed between the DQI and BMI (s.d.) at 6 years. We also considered weight status of the children using definition of overweight or obese according to the International Obesity Task Force cutoffs that are based on BMI;^27^ 140 children (16.0%) were defined as overweight or obese at 6 years. There was no association between the DQI and the risk of being overweight/obese based on BMI (relative risk=0.97 (95% CI: 0.89, 1.06), *P* for trend=0.55; see Supplementary Table S3).

We checked the robustness of our findings in two final analyses. First, we ran the models using only the data from the subgroup of children who had complete FFQ data at all four ages (*n*=725, 39% of all live births). We found similar results, observing inverse associations between DQI and fat mass (s.d.; adjusted *β*=−0.06 (95% CI: −0.10, −0.01), *P* for trend=0.01), but no association was found with BMI (s.d.; adjusted *β*=0.00 (95% CI: −0.04, 0.05), *P* for trend=0.81). Second, we calculated an alternative DQI based on summed values allocated to quarters (rather than thirds) of the distributions of diet quality scores at each age. Using the alternative DQI, similar results were observed to the main analysis, showing inverse associations with fat mass (s.d.; adjusted *β*=−0.04 (95% CI: −0.07, −0.02), *P* for trend=0.002) and no association with BMI (s.d.; adjusted *β*=0.00 (95% CI: −0.04, 0.05), *P* for trend=0.92).

## Discussion

### Main findings

The principal finding of the present study was that the overall quality of the child's diet across early childhood showed strong independent associations with adiposity at age 6 years, whereas associations with BMI were not evident. To our knowledge, the beneficial effects of continued exposure to a diet of higher quality in early childhood on adiposity have not been described before. The differences were large, such that the children with the poorest diets had a fat mass that was 14% greater than those who had diets of highest quality, suggesting that quality of diet established in early childhood may have important implications for obesity risk in later childhood.

### Tracking of diet quality from infancy to early childhood

The tracking of dietary patterns from infancy to childhood has been described in several studies.^18, 28, 29^ For example, in toddlers in an Australian study,^28^ similar dietary patterns reflecting core and non-core food intakes were identified at both 14 and 24 months, whereas in the Avon Longitudinal Study of Parents and Children study consistent dietary patterns have been described at 3, 4 and 7 years of age.^29^ In the present study we observed tracking of a dietary pattern, characterized by frequent consumption of fruits, vegetables and fish, at 6 and 12 months, and 3 and 6 years of age (Spearman's correlation coefficients=0.32–0.69), which suggests a stability in eating habits persisting from infancy to early childhood. As diet quality has been inconsistently linked to differences in body composition in cross-sectional analyses, we created a DQI in this study to represent overall diet quality across early childhood. As observed in Figure 1, by categorizing children according to the DQI we found distinct groups of children with marked differences in diet quality at each age, including a sizeable group (11%), who consistently had the poorest diets and who are of concern.

### Associations of diet quality across early childhood and adiposity

Some observational studies have shown associations between diet quality assessed at multi-time points in childhood and measures of adiposity, although the evidence is inconsistent. For example, in the Raine study, better diet quality at 1 year (but not at 2 or 3 years) was weakly associated with a lower BMI at 5, 8 and 10 years of age, but not at 3 years of age or in adolescence (at 14 or 17 years of age).^30^ Consistent with some previous studies,^10, 30, 31^ the present study showed clear relations between poor diet quality at 6 months and at 3 and 6 years of age, with a higher fat mass at 6 years, although an independent association was not found at 12 months. By creating an index to represent overall diet quality between 6 months and 6 years, our study extended these findings to examine cumulative effects of continued exposure to diets of varying quality during early childhood. Although our findings require replication in other studies, they highlight the importance of diet quality from very early life and the potential for a protective role of diets of better quality in terms of risk of later obesity.

The mechanisms linking variations in the diet quality across early childhood and lower fat mass at 6 years are unknown. One potential mechanism underlying these associations might be that a diet of lower quality is characterized by energy-dense, high-fat foods and a lack of fruits and vegetables, as we observed in the present study. This would be consistent with a recent study showing that a diet high in energy-dense, high-fat and low-fiber foods in childhood or adolescence was associated with later obesity risk,^12^ and may be explained by energy-dense, high-fat foods undermining innate appetite control, leading to greater energy consumption. In comparison, higher dietary fiber intakes increase satiety levels and may delay or decrease subsequent energy intake.^32^ However, although more prospective studies considering cumulative effects of the quality of children's diet are needed to further clarify the role of diet in the etiology of obesity, some insights from the present study are relevant for future interventions. First, the tracking of dietary patterns from 6 months onwards indicates that public health initiatives to improve diet quality of young children need to start very early in life. Second, the evident differences in social background and other health behaviors that are associated with poor diet quality (Table 2) mean that more vulnerable children, for whom greater support is needed, may be readily identifiable in the population. Although changing behavior is challenging, these findings highlight the potential of targeted initiatives to be effective interventions.

In the present study, there were graded associations of dietary patterns and the DQI with body fat mass assessed by DXA measurement, whereas we observed no relations with BMI, used as a surrogate measure of adiposity. The lack of association with BMI is consistent with findings of previous studies,^10, 28, 31^which suggests that BMI may be of limited use as a measure of adiposity in early childhood. As BMI is based on only weight and height, both of which greatly change during growth and development, a high BMI can reflect increase in not only fat mass but also fat-free mass,^13, 33^ indicating that adiposity defined by BMI may be less sensitive than a direct assessment of fat mass, such as DXA, in childhood.

### Strengths and limitations of this study

Strengths of the present study include a prospective design, studying a general population sample of mother–child pairs from a wide range of sociodemographic backgrounds, with comprehensive assessments at multiple time points in pregnancy and childhood, and the use of DXA to provide direct measures of fat mass. However, there are a number of limitations. First, children's body composition measurements were not available for the whole SWS cohort. In comparison with mothers without available children's DXA measurement, the subset of mothers tended to be better educated, less likely to smoke in pregnancy, older and to have breastfed for longer. However, we adjusted for each of these factors in our statistical models, and unless the associations between diet quality scores at each of four ages or the DQI and child's obesity outcomes are different in the remainder of the cohort, it is unlikely that selection bias could explain our findings. Second, although diet at each age was assessed using validated FFQs that were administered by trained research nurses,^14, 15, 16, 17^ misreporting of food intake is a source of measurement error. However, this error would be expected to attenuate associations and we do not think that misreporting on the FFQs could explain the associations we describe. Importantly, FFQs have been shown to identify similar patterns of diet to other dietary assessment methods and dietary pattern scores determined using different methods are highly correlated.^17, 18^ Finally, higher diet-quality scores may act as markers for more healthy behaviors and less obesogenic family influences, which could potentially confound associations with adiposity. Although we controlled for a large number of antenatal and postnatal potential confounding factors and other influences on child adiposity previously described in this cohort,^19, 22, 23, 25^ we cannot rule out unmeasured or residual confounding in an observational study.

In conclusion, an index of overall diet quality from infancy to early childhood was associated with adiposity at the age of 6 years, with the greatest adiposity observed in children who had consistently poorer diets over this period. Our data, together with growing evidence showing that patterns of food intake established in infancy persist to later childhood, suggest that promotion of healthy dietary habits in early life could have an important role as part of public health strategies to prevent childhood obesity and other related chronic diseases in later life.

## Figures and Tables

**Table 1:** Characteristics of 1018 mother–child pairs studied in comparison with the rest of the Southampton Women's Survey cohort

	*Mother–child pairs (*n=*1018)*	*Remaining mother–child pairs (*n=*834)*	P*-value*
*Mother*			
Age at child's birth (years)	30.4 (3.8)	29.7 (3.8)	<0.001
Height (cm)	163.7 (6.5)	162.7 (6.4)	0.001
Prepregnancy BMI (kg m^−2^)	24.3 (22.2–27.5)	24.2 (21.8–28.1)	0.83
Degree qualification or above, *n* (%)	238 (23.5)	151 (18.2)	<0.001
Primiparous, *n* (%)	487 (47.8)	322 (38.7)	<0.001
Smoked in pregnancy, *n* (%)	141 (13.9)	183 (22.2)	<0.001
Serum 25(OH) D concentration (nmol l^−1^)	61.0 (41.3–86.1)	57.0 (41.0–83.0)	0.11
Plasma *n*-6 PUFA concentration (μg ml^−1^)	506 (398–621)	520 (399–633)	0.14
Gestational weight gain, *n* (%)			0.70
Inadequate	206 (21.8)	175 (23.4)	
Adequate	287 (30.3)	218 (29.1)	
Excessive	453 (47.9)	355 (47.5)	
			
*Child*			
Sex, boys, *n* (%)	529 (52.0)	445 (53.6)	0.51
Gestational age (weeks)	40.1 (39.3–41.0)	40.1 (39.2–41.0)	0.59
Birthweight (g)	3520 (476)	3509 (472)	0.65
Duration of breastfeeding (%)			
Never breastfed	159 (15.9)	176 (23.8)	<0.001
<1 Month	211 (21.1)	152 (20.5)	
1–3 Months	193 (19.3)	153 (20.7)	
4–6 Months	182 (18.2)	109 (14.7)	
7–11 Months	157 (15.7)	105 (14.2)	
⩾12 Months	98 (9.8)	45 (6.1)	

Abbreviations: 25(OH) D, 25-hydroxyvitamin D; BMI, body mass index; PUFA, polyunsaturated fatty acid.

Data are presented as mean (s.d.), median (interquartile range) or *n* (percentage). Differences between the two groups of mother–child pair was conducted by *t*-test or Mann–Whitney rank-sum test for continuous variables and *χ*^2^-test for categorical variables.

**Table 2:** Characteristics of 1018 mother–child pairs studied, according to the DQI across early childhood

	*DQI across early childhood*				
	*DQI: 0 (*n=*107)*	*DQI: 1–3 (*n=*339)*	*DQI: 4–6 (*n=*378)*	*DQI: 7–8 (*n=*194)*	P*-value*
*Mother*					
Age at child's birth (years)	29.8 (4.3)	30.0 (3.9)	30.3 (3.7)	31.5 (3.2)	<0.001
Height (cm)	163.1 (5.6)	163.3 (6.5)	163.7 (6.6)	164.5 (6.6)	0.08
Prepregnancy BMI (kg m^−2^)	24.4 (22.2–28.7)	25.0 (22.8–27.7)	24.1 (22.2–27.0)	23.8 (21.6–26.7)	0.003
Degree qualification or above (%)	4 (3.7)	37 (11.0)	102 (27.1)	95 (49.2)	<0.001
Smoked in pregnancy (%)	33 (30.8)	66 (19.5)	35 (9.3)	7 (3.6)	<0.001
Serum 25(OH) D concentration (nmol l^−1^)^a^	58.2 (39.0–81.0)	57.0 (39.0–81.9)	65.0 (42.0–92.0)	65.0 (47.4–91.0)	<0.001
Plasma *n*-6 PUFA concentration (μg ml^−1^)^a^	528 (405–623)	486 (408–616)	525 (408–627)	486 (368–600)	0.81
Gestational weight gain, *n* (%)					0.13
Inadequate	23 (23.2)	66 (20.8)	66 (19.1)	51 (27.7)	
Adequate	24 (24.2)	92 (29.0)	109 (31.5)	62 (33.7)	
Excessive	52 (52.5)	159 (50.2)	171 (49.4)	71 (38.6)	
					
*Child*					
Sex, boys (%)	59 (55.1)	173 (51.0)	195 (51.6)	102 (52.6)	0.86
Height at 6 years (cm)	118.5 (4.8)	119.0 (5.7)	119.6 (5.0)	119.4 (4.8)	0.13
Weight at 6 years (kg)	22.0 (19.9–24.5)	22.5 (20.4–25.1)	22.6 (20.6–25.1)	22.1 (20.7–23.7)	0.43
Birthweight (g)	3406 (464)	3523 (504)	3554 (451)	3510 (470)	0.10
Average time moving at 4 years (h per day)	3.0 (2.0–4.0)	2.0 (1.5–4.0)	2.0 (1.5–3.0)	2.0 (1.0–3.0)	<0.001
Time spending TV at 4 years (h per day)	2.5 (1.5–3.5)	2.5 (1.5–2.5)	1.5 (1.5–2.5)	1.5 (1.5–2.5)	<0.001
Time sleeping per night at 4 years (h per day)	11.3 (11.0–12.0)	11.5 (11.0–11.5)	11.5 (11.0–12.0)	11.5 (11.0–11.5)	0.16
Average time napping at 4 years (h per day)	1.0 (0.0–1.5)	0.5 (0.0–1.0)	0.5 (0.0–0.5)	0.5 (0.0–0.8)	0.21
Duration of breastfeeding⩾4 months (%)	10 (9.4)	118 (35.3)	182 (48.9)	127 (67.6)	<0.001

Abbreviations: 25(OH) D, 25-hydroxyvitamin D; BMI, body mass index; DQI, diet quality index; PUFA, polyunsaturated fatty acid.

Data are presented as mean (s.d.), median (interquartile range) or *n* (percentage).

a Data measured in late pregnancy.

**Table 3:** Associations of diet quality score at each time point of dietary assessment and DQI across early childhood with fat mass *z*-score and BMI *z*-score at 6 years

	*Fat mass at 6 years (s.d.)*						*BMI at 6 years (s.d.)*					
*Diet quality*^a^		*Descriptive*^b^	*Model 1*^c^		*Model 2*			*Descriptive*^b^	*Model 1*^c^		*Model 2*	
	n		β	*95% CI*	β	*95% CI*	n		β	*95% CI*	β	*95% CI*
*At 6 months*												
T1: Low	204	6.0 (5.1–7.5)	0.38	(0.19, 0.56)	0.28	(0.05, 0.51)	269	15.9 (15.0–17.1)	0.18	(0.01, 0.35)	0.12	(−0.10, 0.35)
T2: Medium	221	5.8 (4.8–6.8)	0.15	(−0.03, 0.34)	−0.02	(−0.23, −0.19)	270	15.8 (14.9–16.8)	0.10	(−0.07, 0.27)	−0.05	(−0.26, 0.16)
T3: High	226	5.4 (4.6–6.4)	Reference		Reference		266	15.6 (14.9−16.7)	Reference		Reference	
Effect per 1-unit score increase			−0.17	(−0.25, −0.09)	−0.13	(−0.23, −0.04)			−0.07	(−0.14, 0.00)	−0.04	(−0.14, 0.05)
*P* for trend			<0.001		0.007				0.04		0.36	
												
*At 12 months*												
T1: Low	207	6.0 (5.1–7.3)	0.36	(0.18, 0.55)	0.08	(−0.16, 0.32)	275	15.9 (15.0–16.9)	0.19	(0.02, 0.36)	−0.09	(−0.31, 0.13)
T2: Medium	230	5.8 (4.9–7.0)	0.27	(0.08, 0.45)	0.20	(−0.01, 0.42)	264	15.8 (15.1–17.1)	0.26	(0.09, 0.43)	0.20	(−0.01, 0.40)
T3: High	220	5.5 (4.5−6.6)	Reference		Reference		263	15.5 (14.8–16.5)	Reference		Reference	
Effect per 1-unit score increase			−0.17	(−0.25, −0.09)	−0.04	(−0.14, 0.07)			−0.09	(−0.16, −0.02)	0.04	(−0.06, 0.14)
*P* for trend			<0.001		0.48				0.01		0.43	
												
*At 3 years*												
T1: Low	225	6.1 (5.2–7.6)	0.41	(0.23, 0.59)	0.23	(0.01, 0.45)	298	16.0 (15.1−17.2)	0.27	(0.10, 0.43)	0.07	(−0.15, 0.29)
T2: Medium	241	5.6 (4.7–6.7)	0.05	(−0.13, 0.22)	0.01	(−0.20, 0.21)	291	15.6 (14.9−16.7)	0.05	(−0.11, 0.22)	0.02	(−0.18, 0.23)
T3: High	235	5.5 (4.6–6.5)	Reference		Reference		270	15.6 (14.9−16.5)	Reference		Reference	
Effect per 1-unit score increase			−0.18	(−0.25, −0.10)	−0.12	(−0.22, −0.02)			−0.14	(−0.21, −0.08)	−0.06	(−0.15, 0.04)
*P* for trend			<0.001		0.01				<0.001		0.25	
												
*At 6 years*												
T1: Low	183	6.1 (5.3–7.6)	0.37	(0.17, 0.57)	0.32	(0.08, 0.57)	291	15.8 (14.9−17.0)	0.11	(−0.06, 0.27)	−0.07	(−0.29, 0.15)
T2: Medium	191	5.6 (4.7–6.9)	0.08	(−0.12, 0.28)	0.03	(−0.19, 0.25)	291	15.7 (14.9−16.9)	0.02	(−0.14, 0.18)	−0.06	(−0.26, 0.13)
T3: High	195	5.5 (4.7–6.5)	Reference		Reference		291	15.8 (14.9−16.7)	Reference		Reference	
Effect per 1-unit score increase			−0.15	(−0.23, −0.07)	−0.13	(−0.24, −0.03)			−0.05	(−0.12, 0.02)	0.04	(−0.05, 0.13)
*P* for trend			<0.001		0.01				0.13		0.42	
												
*DQI across early childhood*												
DQI: 0	64	6.1 (5.2–7.6)	0.50	(0.21, 0.79)	0.46	(0.06, 0.86)	88	15.7 (14.7−17.1)	0.22	(−0.04, 0.47)	0.05	(−0.34, 0.43)
DQI: 1–3	237	5.9 (5.1–7.3)	0.49	(0.29, 0.70)	0.28	(0.03, 0.54)	305	15.9 (15.1−17.1)	0.31	(0.12, 0.49)	0.07	(−0.18, 0.31)
DQI: 4–6	274	5.6 (4.7–6.7)	0.20	(0.00, 0.41)	0.13	(−0.11, 0.36)	309	15.8 (15.0−16.8)	0.23	(0.04, 0.41)	0.10	(−0.13, 0.33)
DQI: 7–8	136	5.3 (4.5–6.2)	Reference		Reference		172	15.5 (14.7−16.4)	Reference		Reference	
Effect per 1-unit DQI increase			−0.07	(−0.10, −0.04)	−0.05	(−0.09, −0.01)			−0.04	(−0.07, −0.01)	−0.01	(−0.04, 0.03)
*P* for trend			<0.001		0.01				0.003		0.78	

Abbreviations: 25(OH) D, 25-hydroxyvitamin D; BMI, body mass index; CI, confidence interval; DQI, diet quality index; DXA, dual-energy X-ray absorptiometry; IOM, Institute of Medicine; PCA, principal component analysis; PUFA, polyunsaturated fatty acid.

a Diet quality was determined according to scores for a PCA-defined dietary pattern (that is, infant guidelines pattern at 3 and 6 months and prudent dietary pattern at 3 and 6 years). The distributions of diet quality score at each of the four ages were categorized into thirds.

b Descriptive data was fat mass (kg) and BMI (kg m^−2^).

c Outcome for fat mass adjusted for sex, age at DXA measurements and height; outcome for BMI adjusted for sex and age at measurement. All outcomes were positively skewed and thus were log-transformed. Associations are expressed as regression coefficients (*β*) (95% CI) for standardized variables (*z*-scores; relative change in s.d. of outcome per 1-unit increase of score or index of diet quality). Model 1 is unadjusted model. Model 2 is adjusted for maternal prepregnancy BMI, smoking status in pregnancy, IOM weight-gain categories, maternal serum 25(OH) D concentration in late pregnancy, maternal plasma *n*-6 PUFA concentration in late pregnancy, duration of breastfeeding, time moving each day at 4 years and time spent watching TV at 4 years.
