# Supplementary material for: Diet quality across early childhood and adiposity at 6 years: the Southampton Women's Survey
Source: Int J Obes (Lond). 2015 Jun 30;39(10):1456–62. doi: 10.1038/ijo.2015.97 (PMC4597330; doi:10.1038/ijo.2015.97)
Supplement: Supplementary Tables [file ijo201597x1.doc]

**Supplementary Table S1**. Principal components analysis coefficients for the prudent diet pattern at 6 years

| Food or food group | Coefficient |
| --- | --- |
| Rice and pasta | 0.16 |
| White bread | -0.20 |
| Brown bread | 0.18 |
| Pizza and quiche | 0.04 |
| Breakfast cereals | 0.09 |
| Cakes and biscuits | -0.09 |
| Puddings | 0.03 |
| Full-fat milk (pints) | 0.05 |
| Reduced-fat milk (pints) | 0.02 |
| Yoghurt | -0.02 |
| Cheese and cottage cheese | 0.12 |
| Eggs and egg dishes | 0.17 |
| Spreading fat | -0.03 |
| Red meat | 0.13 |
| Chicken and turkey | 0.11 |
| Offal | 0.00 |
| Processed meat | -0.15 |
| Fish and shellfish | **0.20** |
| Salad vegetables | **0.25** |
| Green vegetables | **0.33** |
| Root vegetables | **0.26** |
| Other vegetables | **0.23** |
| Tinned vegetables | -0.04 |
| Vegetable dishes and vegetarian food | 0.18 |
| Beans and pulses | 0.15 |
| Chips and roast potatoes | -0.22 |
| Boiled potatoes | 0.13 |
| Crisps | -0.23 |
| Crackers | 0.08 |
| Citrus fruit | 0.13 |
| Other fruit | **0.23** |
| Fruit juices | 0.12 |
| Dried fruit | 0.14 |
| Cooked and tinned fruit | 0.09 |
| Nuts and seeds | 0.18 |
| Added sugar (teaspoons) | -0.09 |
| Sweet spreads | 0.03 |
| Confectionery | -0.14 |
| High-energy soft drinks | -0.03 |
| Low calorie soft drinks | -0.19 |
| Tea and coffee | -0.06 |
| Milky drinks | 0.02 |
| Miscellaneous | 0.15 |
| Sauces and salad dressings | 0.07 |
| Water | 0.17 |
| Marmite and Bovril | 0.05 |
|  |  |
| Percentage of variation explained | 7.5% |

**Supplementary Table S2**. Correlation matrix of the infant guidelines/prudent diet scores at each of the four ages

|  | Infant guidelines/prudent diet score | | | |
| --- | --- | --- | --- | --- |
| Infant guidelines /prudent diet score | 6 months | 12 months | 3 years | 6 years |
| 6 months | 1.00 | - | - | - |
| 12 months | 0.53 | 1.00 | - | - |
| 3 years | 0.38 | 0.63 | 1.00 | - |
| 6 years | 0.32 | 0.51 | 0.69 | 1.00 |

Spearman correlation coefficient (*r*) of infant guidelines/prudent diet scores between ages assessed (*P*-all correlations <0.001).

**Supplementary Table S3**. Risk of overweight/obesity among children according to the diet quality score at each time point of dietary assessment and DQI across early childhood

|  | Risk of overweight/obesity defined by IOTF | | | | | | |
| --- | --- | --- | --- | --- | --- | --- | --- |
| Diet quality ^a^ | *n* | overweight /obese ^b^ | Model 1 | |  | Model 2 | |
|  |  |  | RR | 95% CI |  | RR | 95% CI |
| At 6 months |  |  |  |  |  |  |  |
| T1: Low | 269 | 52 | 1.66 | (1.10, 2.50) |  | 1.56 | (0.86, 2.82) |
| T2: Medium | 270 | 47 | 1.49 | (0.98, 2.28) |  | 1.39 | (0.80, 2.41) |
| T3: High | 266 | 31 | Reference | |  | Reference | |
| Effect per 1-unit score increase | | | 0.86 | (0.73, 1.01) |  | 0.94 | (0.72, 1.21) |
| *P* for trend |  |  | 0.07 | |  | 0.62 | |
|  |  |  |  |  |  |  |  |
| At 12 months |  |  | | |  |  | |
| T1: Low | 275 | 44 | 1.45 | (0.94, 2.25) |  | 0.88 | (0.49, 1.58) |
| T2: Medium | 264 | 53 | 1.82 | (1.20, 2.77) |  | 1.47 | (0.86, 2.52) |
| T3: High | 263 | 29 | Reference | |  | Reference | |
| Effect per 1-unit score increase | | | 0.86 | (0.74, 1.01) |  | 1.15 | (0.91, 1.44) |
| *P* for trend |  |  | 0.07 | |  | 0.24 | |
|  |  |  |  |  |  |  |  |
| At 3 years |  |  | | |  |  | |
| T1: Low | 298 | 63 | 1.84 | (1.24, 2.74) |  | 1.09 | (0.64, 1.87) |
| T2: Medium | 291 | 43 | 1.29 | (0.84, 1.98) |  | 1.07 | (0.62, 1.83) |
| T3: High | 270 | 31 | Reference | |  | Reference | |
| Effect per 1-unit score increase | | | 0.73 | (0.63, 0.84) |  | 0.91 | (0.73, 1.14) |
| *P* for trend |  |  | <0.001 | |  | 0.42 | |
|  |  |  |  |  |  |  |  |
| At 6 years |  |  | | |  |  | |
| T1: Low | 291 | 58 | 1.49 | (1.03, 2.16) |  | 0.99 | (0.61, 1.62) |
| T2: Medium | 291 | 42 | 1.08 | (0.72, 1.61) |  | 0.97 | (0.58, 1.60) |
| T3: High | 291 | 39 | Reference | |  | Reference | |
| Effect per 1-unit score increase | | | 0.80 | (0.68, 0.93) |  | 0.94 | (0.74, 1.18) |
| *P* for trend |  |  | 0.005 | |  | 0.59 | |
|  |  |  |  |  |  |  |  |
| Diet quality index across early childhood | | | | |  | |  |
| DQI: 0 | 88 | 17 | 1.75 | (0.96, 3.19) |  | 0.96 | (0.39, 2.34) |
| DQI: 1-3 | 305 | 59 | 1.75 | (1.08, 2.84) |  | 1.01 | (0.54, 1.88) |
| DQI: 4-6 | 309 | 45 | 0.32 | (0.80, 2.18) |  | 0.88 | (0.47, 1.63) |
| DQI: 7-8 | 172 | 19 | Reference | |  | Reference | |
| Effect per 1-unit DQI increase | | | 0.91 | (0.85, 0.96) |  | 0.97 | (0.89, 1.06) |
| *P* for trend |  |  | 0.002 | |  | 0.55 | |

Abbreviations: DQI, diet quality index; IOTF, International Obesity Task Force.

^a^ Diet quality was determined according to scores for a principal component analysis-defined dietary pattern (i.e. infant guidelines pattern at 3 and 6 months and prudent dietary pattern at 3 and 6 years). The distributions of diet quality scores at each of the four ages were categorized into thirds. ^b^ Overweight or obese was defined according to the IOTF cut offs (ref. 27). Relative risks calculated using Poisson regression with robust variance. Model 1 is unadjusted model. Model 2 adjusted for maternal prepregnancy BMI, smoking status in pregnancy, IOM weight-gain categories, maternal serum 25-hydroxy vitamin D concentration in late pregnancy, maternal plasma n-6 polyunsaturated fatty acid concentration in late pregnancy, duration of breast-feeding, time moving each day at 4 years and time spent watching TV at 4 years.
